# Interleukin 17A: a Janus-faced regulator of osteoporosis

**DOI:** 10.1038/s41598-020-62562-2

**Published:** 2020-03-30

**Authors:** J. M. Scheffler, L. Grahnemo, C. Engdahl, C. Drevinge, K. L. Gustafsson, C. Corciulo, L. Lawenius, Y. Iwakura, K. Sjögren, M. K. Lagerquist, H. Carlsten, C. Ohlsson, U. Islander

**Affiliations:** 1 Krefting Research Center, Department of Internal Medicine and Clinical Nutrition, Institute of Medicine, Sahlgrenska Academy at University of Gothenburg, Gothenburg, Sweden; 2 Centre for Bone and Arthritis Research, Department of Internal Medicine and Clinical Nutrition, Institute of Medicine, Sahlgrenska Academy at University of Gothenburg, Gothenburg, Sweden; 3 Centre for Bone and Arthritis Research, Department of Rheumatology and Inflammation Research, Institute of Medicine, Sahlgrenska Academy at University of Gothenburg, Gothenburg, Sweden; 4 Department of Drug Treatment, Sahlgrenska University Hospital, Gothenburg, Sweden; 5 Research Institute for Biomedical Sciences, Tokyo University of Science, Chiba, Japan

**Keywords:** Endocrinology, Experimental models of disease, Osteoimmunology

## Abstract

Interleukin (IL)-17A is a well-described mediator of bone resorption in inflammatory diseases, and postmenopausal osteoporosis is associated with increased serum levels of IL-17A. Ovariectomy (OVX) can be used as a model to study bone loss induced by estrogen deficiency and the role of IL-17A in osteoporosis development has previously been investigated using various methods to inhibit IL-17A signaling in this model. However, the studies show opposing results. While some publications reported IL-17A as a mediator of OVX-induced osteoporosis, others found a bone-protective role for IL-17 receptor signaling. In this study, we provide an explanation for the discrepancies in previous literature and show for the first time that loss of IL-17A has differential effects on OVX-induced osteoporosis; with IL-17A being important for cortical but not trabecular bone loss. Interestingly, the decrease in trabecular bone after OVX in IL-17A knock-out mice, was accompanied by increased adipogenesis depicted by elevated leptin levels. Additionally, the bone marrow adipose tissue expanded, and the bone-turnover decreased in ovariectomized mice lacking IL-17A compared to ovariectomized WT mice. Our results increase the understanding of how IL-17A signaling influences bone remodeling in the different bone compartments, which is of importance for the development of new treatments of post-menopausal osteoporosis.

## Introduction

Bone homeostasis is an orchestrated process involving both osteoblasts (OBL), which produce new bone, and osteoclasts (OCL) that break it down. While OBL are mono-nucleated cells originating from mesenchymal stem cells (MSC), OCL are multinucleated cells arising from the hematopoietic lineage by fusion of OCL precursor cells (pOCL)^1,2^. This fine-tuned equilibrium of bone formation and resorption can easily be set off-balance in response to hormonal changes, inflammation, and growth.

Reduced levels of endogenously produced estrogens are the major cause of osteoporosis in post-menopausal women and hormone replacement therapy is known to prevent fractures^3,4^. However, long-term treatment with estradiol (E2) can result in severe side effects including certain malignancies and is therefore not recommended anymore^5^. E2 plays a critical role in the regulation of bone mass homeostasis by controlling bone-forming OBL and bone-resorbing OCL. In post-menopausal women, the balance between bone formation and resorption is skewed towards resorption, resulting in a reduction of bone mineral density (BMD). E2 protects bone directly through two main mechanisms. First, it induces osteoprotegrin (OPG) expression in OBL. OPG serves as a decoy receptor for the cytokine receptor activator of NFκB ligand (RANKL), the primary differentiation factor for OCL^6^. Second, E2 protects bone through induction of OCL apoptosis via autocrine Fas-ligand signaling^7^. Furthermore, E2 affects the bone marrow (BM) composition and leads to a decrease in B lymphocyte numbers, a cell type known to be a major producer of RANKL^8^. Thus, in addition to its direct effects on OBL and OCL, E2 also influences the immune system. Recent literature suggests that the increase in bone resorption after menopause is largely mediated by changes in cytokine production by the bone microenvironment as a response to reduced estrogen levels. In addition to an increase in RANKL expression, elevated levels of the pro-inflammatory cytokines tumor necrosis factor alpha and IL-1β were also observed in post-menopausal women, and treatment with the corresponding inhibitors showed a promising reduction in bone resorption^9–12^.

Post-menopausal women suffering from osteoporosis have also been shown to have elevated serum concentrations of IL-17A, which correlates negatively with BMD^13^. In addition, IL-17A levels are elevated in many inflammatory diseases associated with osteoporosis, *e.g*. periodontitis and rheumatoid arthritis (RA)^14^. IL-17A mediates bone pathology by upregulating the relative ratio of RANKL to OPG, which promotes osteoclastogenesis^15^. Further, it was shown that OVX of mice results in an increase in IL-17A producing T helper 17 cells in BM, and that treatment with neutralizing IL-17 antibodies protect against OVX-induced bone loss^16–18^. An additional study confirmed that also mice lacking the principal IL-17 receptor (IL-17RA) or its downstream effector protein, Act1, are protected from the skeletal effects of OVX^19^. Conversely, another group demonstrated that IL-17RA knockout mice have increased susceptibility to OVX-induced bone loss^20^.

Therefore, the specific role of IL-17A in OVX-induced bone loss still remains unclear. To further elucidate the impact of IL-17A on bone homeostasis, we thoroughly investigated the consequences of OVX on different bone compartments, as well as on osteoclastogenesis, in IL-17A knockout (KO) mice^21^. The results show that IL-17A is important for cortical but not trabecular bone loss induced by estrogen deficiency. Furthermore, we demonstrate that IL-17A regulates adipogenesis after OVX resulting in an increase of BM adipose tissue (BMAT). These data provide new evidence that IL-17A has different regulatory effects on trabecular bone, as compared to cortical bone, possibly by affecting adipogenesis in the absence of estrogen.

## Material and methods

### Mice

All experimental procedures were approved by the Ethics Committee at the University of Gothenburg and carried out in accordance with relevant guidelines. The mice were housed in a standard animal facility under controlled temperature (22 °C) and photoperiod (12 h of light and 12 h of darkness). They were fed a pellet diet (Teklad diet 2016, Envigo) and tap water *ad libitum*. The IL-17A knockout (KO) mice were provided by Professor Yoichiro Iwakura (Tokyo University of Science, Japan) and were described before^21^. The deletion of IL-17A was confirmed on protein level in supernatants from stimulated lymph node (LN) cell cultures using ELISA (Supplementary Fig. 1a).

Nine-week-old female KO and littermate wildtype control mice (WT) were ovariectomized (OVX) or sham-operated. Surgery was performed under anesthesia with isoflurane (Baxter Medical AB, Kista, Sweden), and Rimadyl (Orion Pharma AB, Animal Health, Sollentuna, Sweden) was given preoperatively as an analgesic. At termination, three weeks post-surgery, mice were anesthetized with Ketanest/Dexdomitor (Pfizer/Orion Pharma AB), bled and sacrificed by cervical dislocation. Uterus and gonadal fat pads were collected and weighed. One femur and tibia were dissected and fixed in 4% formaldehyde and stored for further analysis. From the other tibia, BM was flushed and bone as well as BM were snap-frozen for RNA analysis. The second femur was collected for flow cytometry analysis. Pooled data from two independently performed experiments (n = 5–8 per group in each experiment) are presented in the main figures. Individual results from the two experiments are shown in the supplementary figures.

### Flow cytometry

BM cells from femur were flushed out and erythrocytes were lysed. The remaining cells were stained with eBioscience Fixable Viability Dye eFluor 780 (Thermo Fisher Scientific), followed by incubation with Fc-gamma receptor block (Becton Dickinson (BD)). Afterwards, the cells were stained with the following fluorescent-labeled antibodies (Biolegend): CD11b-BrilliantViolet (BV) 421, CD19-BV421, CD8-BV510, CD11c-FITC, RANK- Phycoerythrin (PE), RANKL-PE, F4/80-PE Cyanine7 (Cy7), CD3-PECy7, Gr-1-Peridinin-Chlorophyll-Protein (PerCP), MCSF-R-Allophycocyanin (APC), CD4-APC. Fluorescence minus one (FMO) stained samples were used as controls. The cells were either immediately acquired or fixed in 4% paraformaldehyde, before acquisition using FACSVerse (BD). The data were analyzed with the FlowJo software Version 10 (FlowJo).

### Dual-energy X-ray absorptiometry

Analysis of total body BMD and percentage of body fat was performed using a Lunar PIXImus mouse densitometer (Wipro GE Healthcare) for the first experiment and a Faxitron UltraFocus dual-energy x-ray absorptiometry (Faxitron Bioptics, Tuscon, AZ, USA) for the second experiment. The region of interest in the spine was defined as vertebra L3 until L6.

### Peripheral quantitative computed tomography (pQCT)

Computed tomographic scans were performed with the pQCT XCT RESEARCH M (version 4.5B, Norland, Fort Atkinson, WI, USA) operating at a resolution of 70 μm, as described previously^22^. Trabecular bone in the distal femur was analyzed *ex vivo* in a metaphyseal scan and defined as the inner 45% of the total cross-sectional area.

### High-resolution microCT (µCT)

High-resolution µCT analyses were performed using Skyscan 1172 scanner (Bruker MicroCT, Aartselaar, Belgium) as previously described^23^. Briefly, femur was imaged with an X-ray tube voltage of 50 kV, a current of 200 µA, and a 0.5 mm aluminum filter. The scanning angular rotation was 180°, and the angular increment was 0.70°. The voxel size was 4.49 µm isotropically. NRecon (version 1.6.9) was used to perform the reconstruction after the scans. In femur, the trabecular bone proximal to the distal growth plate was selected for analyses within a conforming volume of interest (cortical bone excluded) commencing at a distance of 650 µm from the growth plate, and extending a further longitudinal distance of 134 µm in the proximal direction. Cortical measurements were performed in the diaphyseal region of the femur starting at a distance of 5234 µm from the growth plate and extending a further longitudinal distance of 134 μm in the proximal direction.

### ELISA for IL-17A and IL-17F expression

Single-cell suspensions from inguinal lymph nodes and BM were stimulated with either concanavalin A (ConA, 1.25 µg/ml) or ionomycin calcium salt (1 μg/ml, Sigma)/ phorbol 12-myristate 13-acetate (PMA, 50 ng/ml, Sigma) for 48 h. Supernatants were collected and IL-17A (eBioscience) and IL-17F levels (Invitrogen) were measured according to the manufacturer’s instructions.

### ELISA for serum biomarkers

Blood samples were collected at two and three weeks after surgery. The bone resorption marker C-terminal type I collagen fragments were obtained using an ELISA RatLaps kit (CTX-I, Immunodiagostic Systems). Procollagen type I N propeptide (PINP, Immunodiagostic Systems) was analyzed as a marker of bone formation. Additionally, serum leptin levels were assessed using an ELISA kit (Invitrogen, Thermofisher Scientific). All ELISA assays were performed according to the manufacturer’s instructions.

### qPCR

RNA from cortical bone (tibia) and BM was extracted using TriZol Reagent (Sigma) followed by RNeasy Mini QIAcube kit (Qiagen). qPCR was run using the StepOnePlus Real-Time PCR systems (Applied Biosystems). Predesigned probes for *Tnfsf11 (Rankl)* (Mm00441908_m1) and *Adipoq (adiponectin)* (Mm00448870_cn) were used from Applied Biosystems. The mRNA abundance of each gene was calculated using the “ΔΔCt method” and adjusted for expression of 18 S ribosomal RNA (4310893E, Applied Biosystems).

### Histology of femur

Femurs were fixed in 4% formaldehyde for two days, decalcified with 10% EDTA and embedded in paraffin. 5 µm longitudinal sections were stained with hematoxylin and tartrate-resistant acid phosphatase (TRAP), as previously described^24^. Pictures were acquired with a Nikon Eclipse 80i 124 microscope. Quantifications for OCL and BM adipocytes were done using the Osteomeasure software (v.3.2.1.7; Osteometrics). In detail, OCL numbers were quantified on the diaphyseal endosteal bone surface starting 3 mm distal from the proximal growth plate and continuing for 6.9 mm. In the proximal femur, starting from the growth plate and continuing 2.4 mm in the distal direction, bone marrow adipocytes were identified as circular or semicircular areas devoid of staining, and then bone marrow adiposity (adipocyte area/marrow area; %), adipocyte density (number of adipocytes/marrow area; 1/mm^2^), adipocyte size (mm^2^), and bone marrow area were determined.

### Calcein labelling

Mice were injected with Calcein (Sigma) twice: eight days and one day before sacrifice. Femur longitudinal sections were analyzed using an SP8 confocal microscope equipped with a 488 blue laser and HyD detectors (Leica). Pictures of the double Calcein incorporations on the diaphyseal endosteal bone were taken and distances were measured with the LAS X SP8 software (Leica). 2–3 pictures per sample were analyzed and 15 measurements in total per sample were taken.

### Statistical analyses

Statistical analyses were performed using SPSS software 21.0 (IBM, Armonk, NY, USA) and GraphPad Prism (version 7.03). Each individual experiment was terminated on two succeeding days and variation between days was therefore assessed and corrected for when needed using univariate general linear model. Results are presented as mean ± SEM. Normal distribution was tested with the Shapiro-Wilk normality test. In the case of normal distribution, a one-way ANOVA followed by Tukey’s post hoc test was used for comparisons between all groups. Otherwise, the data was tested with the Kruskal Wallis test followed by Dunn’s post hoc test. Student’s t-test was used for comparison of two independent groups. p < 0.05 was considered significant.

## Results

### IL-17A triggers cortical but not trabecular bone loss after OVX

**Figure 1:**
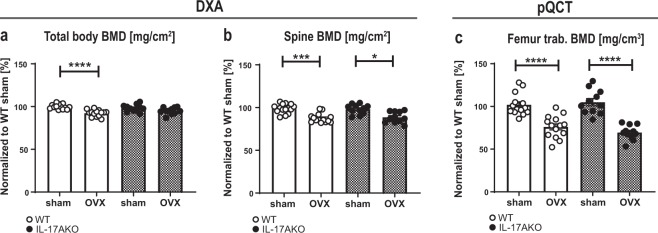
Interleukin (IL)-17A is not important for ovariectomy (OVX) -induced trabecular bone loss. Female wildtype (WT) and IL-17A (KO) mice were subjected to OVX or sham surgery at nine weeks of age and sacrificed three weeks later. (**a**) Total body bone mineral density (BMD) and (**b**) spine BMD was measured three weeks after surgery by dual-energy X-ray absorptiometry (DXA). (**c**) After sacrifice, trabecular BMD was analyzed in femur by peripheral quantitative computed tomography (pQCT). (**a–c**) Results from two independently performed experiments are depicted as the change in percentage from the sham WT group where the mean from each experiment was set to 100%. n = 15 WT sham females; 14 WT OVX females; 11 KO sham females; 11 KO OVX females. *P < 0.05, ***P  < 0.001, ****P < 0.0001, One-way ANOVA. Each dot represents one mouse.

**Figure 2:**
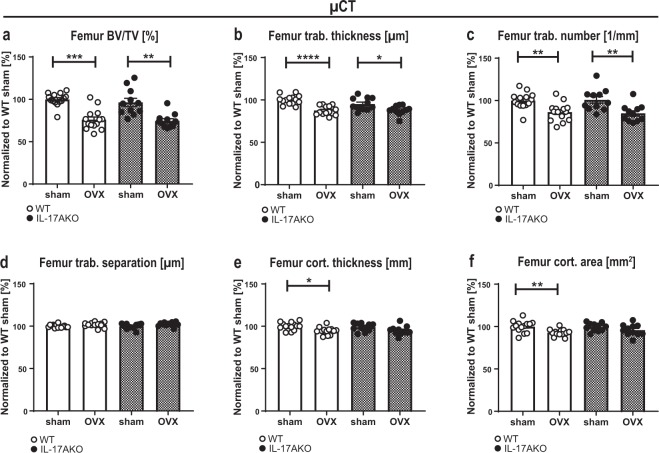
IL-17A is important for OVX-induced cortical but not trabecular bone loss. Female WT and KO mice were subjected to OVX or sham surgery at nine weeks of age and sacrificed three weeks later. Femurs were subjected to high-resolution μ-computed tomography (µCT). (**a**) Trabecular bone volume fraction (bone volume over total volume (BV/TV)), (**b**) trabecular thickness, (**c**) trabecular number, (**d**) trabecular separation, (**e**) cortical thickness and (**f**) cortical area were measured. (**a–f**) Results from two independently performed experiments are depicted as the change in percentage from the sham WT group where the mean from each experiment was set to 100%. n = 15 WT sham females; 14 WT OVX females; 11 KO sham females; 11 KO OVX females. *P < 0.05, **P  < 0.01, ***P < 0.001, One-way ANOVA. Each dot represents one mouse.

Female WT and KO mice underwent OVX at the age of nine weeks and were sacrificed three weeks later. As expected, uterus weight decreased in both genotypes after OVX (Supplementary Fig. 1b), confirming successful removal of the ovaries. At sacrifice, total BMD was analyzed using DXA. The total body BMD of the WT mice decreased significantly after OVX, whereas the BMD of the KO mice remained constant (Fig. 1a). In the spine, however, the BMD of vertebras L3 to L6 decreased in both genotypes after OVX (Fig. 1b, Supplementary Fig. 2a,b). Furthermore, dissected femurs were analyzed by pQCT. The trabecular BMD declined in both the ovariectomized WT and KO mice (Fig. 1c, Supplementary Fig. 2c). Next, both trabecular and cortical bone parameters were quantified in higher resolution using µCT. Trabecular bone volume as a percentage of tissue volume (BV/TV) (Fig. 2a), trabecular thickness (Fig. 2b) and trabecular number (Fig. 2c) decreased in both WT and KO mice after OVX, whereas trabecular separation was unaffected by OVX (Fig. 2d). Cortical thickness (Fig. 2e) and cortical area (Fig. 2f) were both significantly reduced in WT mice following OVX. Interestingly, this effect was absent in KO mice (Supplementary Fig. 3a–f). In summary, the data demonstrate that OVX-induced trabecular bone loss is not dependent on IL-17A. In contrast, IL-17A is important for mediating cortical bone loss after OVX.

### IL-17A induces osteoclastogenesis and supports bone turnover after OVX

**Figure 3:**
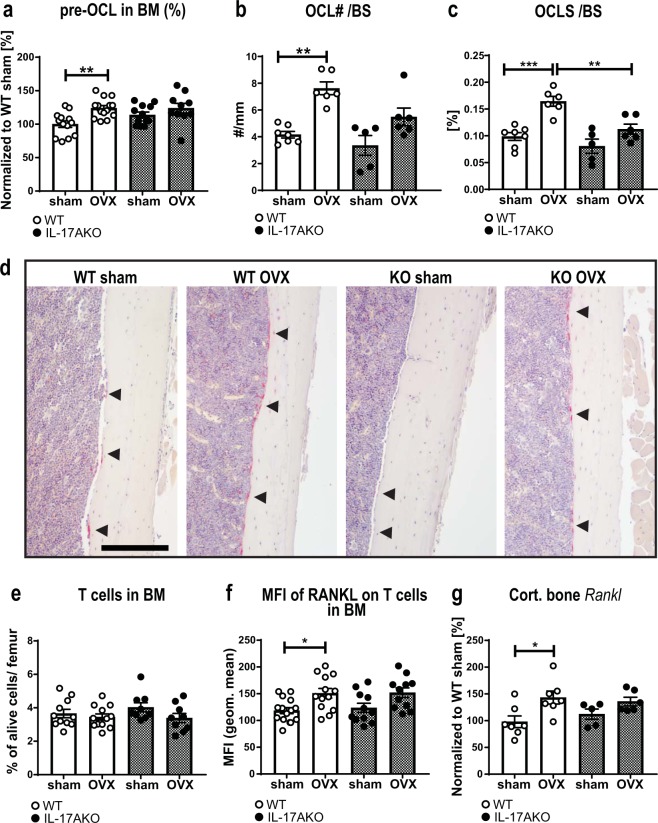
OVX-induced increase of osteoclastogenesis is mediated by IL-17A. Female WT and KO mice were subjected to OVX or sham surgery at nine weeks of age and sacrificed three weeks later. At termination, bone marrow (BM) cells from femur were analyzed with flow cytometry. (**a**) RANK^+^ MCSF-R^−^ pre-osteoclasts (pOCL) were gated from alive cells. Femur was sectioned and stained with hematoxylin (blue) and TRAP (red) to visualize OCL on the diaphyseal endosteal bone surface (BS). Results are presented as (**b**) OCL number (OCL#) per BS and (**c**) OCL surface (OCLS) per BS. (**d**) Representative image of paraffin sections from the diaphyseal region of the femur. Arrowheads indicate areas with OCL. Scale = 200 µm. (**e**) Flow cytometry analysis of CD3^+^ T cells in BM. (**f**) Mean fluorescent intensity (geometric mean) of RANKL on alive CD3^+^ T cells. (**g**) *Rankl* expression in cortical bone (tibia) was analyzed by qPCR and percentage of WT sham are shown. (**a**,**g)** Results from two independently performed experiments are depicted as the change in percentage from the sham WT group where the mean from each experiment was set to 100%. (**a,e,f**) n = 14 WT sham females; 13 WT OVX females; 11 KO sham females; 11 KO OVX females. (**b,c,g)** n = 7 WT sham females; 6–7 WT OVX females; 5 KO sham females; 6 KO OVX females. *P < 0.05, **P  < 0.01, One-way ANOVA. Each dot represents one mouse.

Given the differential effects observed in trabecular and cortical bone compartments after OVX, we next investigated osteoclastogenesis in BM by flow cytometry. Total BM cell number in femur did not differ between genotypes or treatment groups (Supplementary Fig. 4a). The frequency of pOCL increased after OVX in WT but not in KO mice (Fig. 3a, Supplementary Fig. 4b,c). The results were confirmed by quantification of OCL on the diaphyseal endosteal bone surface of femur. The OCL number and OCL surface were significantly increased in WT but not in KO mice after OVX (Fig. 3b–d, black arrows indicate OCL (red)).

**Figure 4:**
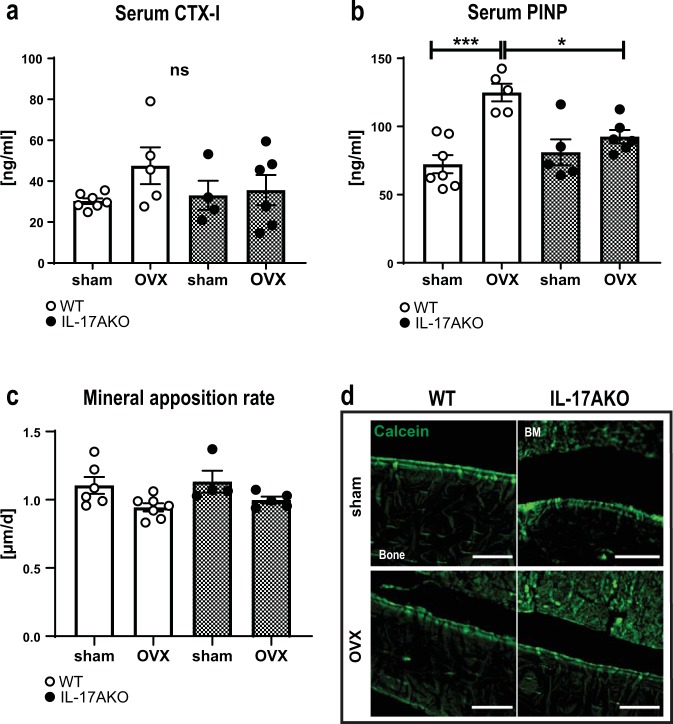
IL-17A is important for the OVX-induced increase in bone formation. Female WT and KO mice were subjected to OVX or sham surgery at nine weeks of age. (**a**) The bone degradation marker C-terminal telopeptide of type I collagen (CTX-I) and (**b**) the bone formation marker N-terminal propeptide of type I procollagen (PINP) were measured in serum from female mice 2 weeks after surgery. (**c**) The mineral apposition rate of cortical bone in femur was analyzed by measuring the distance between the calcein (green) incorporation lines depicted on representative images in (**d**). Scale 100 µm. n = 6–7 WT sham females; 5–7 WT OVX females; 4–5 KO sham females; 5–6 KO OVX females. *P < 0.05, **P  < 0.01, One-way ANOVA. Each dot represents one mouse.

Additional analyses of lymphocyte populations in BM were performed, and in keeping with previously published results, the percentage of B cells increased in WT mice after OVX (Supplementary Fig. 4d)^25^, whereas the T cell population remained unaffected (Fig. 3e). Interestingly, the B cell compartment increased also in KO mice after OVX (Supplementary Fig. 4d). Both B and T cells are known producers of RANKL^26^, therefore surface expression of RANKL was measured. Following OVX, RANKL expression increased on WT T cells, and trended upward also on KO T cells (Fig. 3d, Supplementary Fig. 5). No RANKL expression on B cells could be detected in our setting. In a second approach, mRNA expression of *Rankl* in cortical bone was assessed using qPCR. Similar to the results on T cells, the expression of *Rankl* in cortical bone increased significantly after OVX in WT but not in KO mice (Fig. 3e). Additionally, bone turnover markers in serum of mice were measured at two weeks post-surgery. While C-telopeptide of type I collagen (CTX-I) is a marker for bone resorption and N-terminal propeptide of type I procollagen (PINP) is a bone formation marker, both markers are known to increase up to two weeks after OVX^27^. CTX-1 levels trended upward and PINP serum levels were significantly increased in ovariectomized WT mice. In contrast, neither CTX-1 nor PINP were increased after OVX in KO mice (Fig. 4a,b). Furthermore, the mineral apposition rate in the diaphyseal endosteal region of the femur was assessed by calcein labelling. No significant differences were observed between the groups, however, a tendency towards less bone formation after OVX in both WT and KO mice was observed (Fig. 4c,d). Together, these data support the conclusion that IL-17A increases bone resorption and might also affect bone formation after OVX.

### IL-17A inhibits adipogenesis of BM fat after OVX

**Figure 5:**
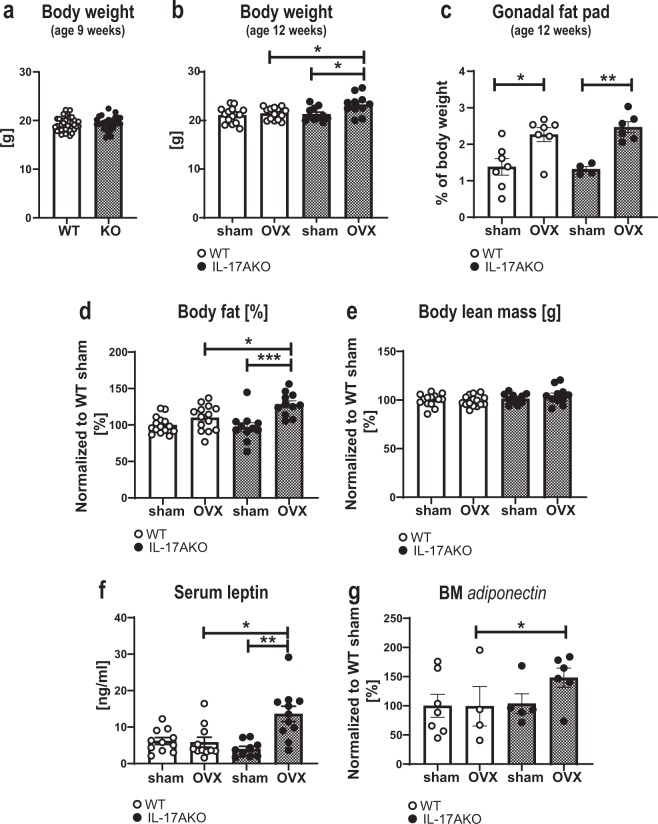
IL-17A suppresses OVX-induced increase in adipogenesis. Female WT and KO mice were subjected to OVX or sham surgery at nine weeks of age and sacrificed three weeks later. (**a**) Body weight of female WT and KO mice at nine weeks of age. (**b**) Body weight at the termination of the experiments (three weeks post-surgery, 12 weeks of age). (**c**) Gonadal fat pad weight as % of total body weight. (**d**) Body fat mass as percentage of body weight measured by DXA at the termination of the experiments. (**e**) Body lean mass measured by DXA. (**f**) Serum levels of leptin assessed by ELISA. (**f**) *Adiponectin* expression in BM analyzed by qPCR and percentage of WT sham are shown. (**d,e,g)** Results from two independently performed experiments are depicted as change in percentage from the sham WT group where the mean from each experiment was set to 100%. (**a**) n = 29 WT females; 22 KO females, Students t-test. (**c,g)** n = 7 WT sham females; 4–7 WT OVX females; 4–5 KO sham females; 6 KO OVX females. (**b,d,e,f**) n = 15 WT sham females; 14 WT OVX females; 11 KO sham females; 11 KO OVX females. *P < 0.05, **P < 0.01, ***P < 0.001, One-way ANOVA. Each dot represents one mouse.

**Figure 6:**
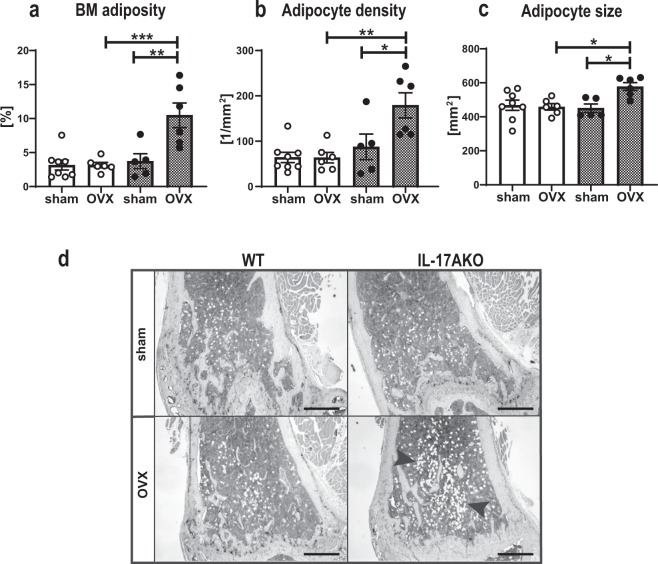
IL-17A regulates BM adipogenesis after OVX. Female WT and KO mice were subjected to OVX or sham surgery at nine weeks of age and sacrificed three weeks later. Femur was sectioned and stained with hematoxylin and TRAP. Adipocytes were quantified and results are shown as (**a**) BM adiposity (adipocyte area/marrow area; %) (**b**) adipocyte density (number of adipocytes/marrow area; 1/mm^2^) and (**c**) adipocyte size (mm^2^). (**d**) Representative paraffin sections of the metaphyseal region of femur are shown, the color is reduced to greyscale to visualize adipocytes (white). Arrowheads indicate areas with adipocytes. One representative image is shown. Scale = 500 µm. (**a–c**) n = 8 WT sham females; 6 WT OVX females; 5 KO sham females; 6 KO OVX females. *P < 0.05, **P < 0.01, ***P < 0.001, One-way ANOVA. Each dot represents one mouse.

IL-17 has been demonstrated to exert inhibitory effects on adipogenesis, and IL-17 KO mice develop more severe obesity in the setting of high fat feeding compared to WT mice^28^. Before OVX (at nine weeks of age), the body weight did not differ between WT and KO mice (Fig. 5a). Three weeks after OVX (at twelve weeks of age), the body weight did not differ between ovariectomized or sham-operated WT mice, while ovariectomized KO mice had increased body weight compared with sham KO mice (Fig. 5b). Interestingly, the gonadal fat pad weight of both genotypes increased significantly after OVX (Fig. 5c). Additionally, the percentage of body fat measured by DXA only increased in the KO mice post-surgery (Fig. 5d, Supplementary Fig. 6a) while the lean body mass did not vary between groups (Fig. 5e, Supplementary Fig. 6b). Leptin and adiponectin are markers secreted from adipose tissue, and leptin secretion by adipocytes is proportional to body fat stores^29^. Serum levels of leptin remained constant after OVX in WT mice, but were elevated in ovariectomized KO mice (Fig. 5f). Furthermore, ovariectomized KO mice had increased *adiponectin* expression compared to ovariectomized WT mice, while there was no difference after OVX in WT mice (Fig. 5g). Finally, histological examinations of BM adipocytes in the metaphyseal region of the femur showed increased levels of BM adiposity, adipocyte density and adipocyte size in the KO mice after OVX, while no differences were detected in WT mice (Fig. 6a–d, black arrows and Supplementary Fig. 7). In summary, these data suggest a role for IL-17A in adipogenesis following OVX.

## Discussion

This work provides new evidence that IL-17A regulates bone homeostasis after OVX differentially in cortical and trabecular bone. While the deletion of IL-17A protects mice from OVX-induced cortical bone loss, trabecular bone loss is similar to that of ovariectomized WT mice. Interestingly, we observe an increase in adipogenesis in KO mice after OVX, which could affect bone remodeling.

Previous publications have shown that bone loss after OVX is associated with increased number of IL-17A secreting Th17 cells in BM. Furthermore, the addition of IL-17A to co-cultures of OCL and OBL was shown to increase the secretion of RANKL by OBL and stimulate osteoclastogenesis to a higher extent, compared with cultures without the addition of IL-17A^17^. Finally, the treatment of mice with an IL-17 antibody was shown to be protective against OVX-induced bone loss^16,30^. In line with these results, a publication from DeSelm *et al*. showed that abrogation of IL-17 signaling by deletion of either the IL-17RA receptor or the downstream signaling component Act1 also protects mice from OVX-induced osteoporosis^31^. Collectively, these publications report that the ablation of IL-17A signaling protects the skeleton after OVX. However, in contrast to these studies, Goswami *et al*. showed that the deletion of IL-17RA aggravates OVX-induced bone loss, thus showing a bone-protective role for IL-17A receptor signaling^20^. IL-17A is secreted by many immune cells including Th17 cells, gamma delta T cells, natural killer T cells and type 3 innate lymphoid cells^32,33^, and in a recent publication, we showed that estrogen receptor alpha signaling in T cells is dispensable in mediating OVX-induced bone loss^34^. Therefore, to further investigate the discrepancy in the literature on effects of IL-17 in OVX-induced bone loss we used a knock-out mouse model where IL-17A is deleted globally^35^ and thoroughly analyzed the effects of OVX in different compartments of the skeleton.

In accordance with the majority of previously published literature, we found using the DXA technique, that total body BMD of mice lacking IL-17A was unaffected by OVX, while lumbar spine BMD in KO mice was reduced by OVX to a similar extent as WT mice. Importantly, total body BMD measured by DXA reveals the combined BMD of both cortical and trabecular bone compartments, whereas spine BMD mainly reflects trabecular bone. Interestingly, the differing effects on total body BMD and spine BMD after OVX in KO mice indicates that IL-17A could have different effects on cortical and trabecular bone.

Further detailed analyses of cortical and trabecular bone compartments using pQCT and µCT confirmed this hypothesis and showed that lack of IL-17A protects from OVX-induced cortical but not trabecular bone loss. This is an important new finding shedding light on the discrepancy between previously published papers. To understand the mechanisms involved in these differential effects of IL-17A on OVX-induced cortical and trabecular bone loss, we analyzed the phenotype of the KO mice in more detail.

Osteoporosis is associated with increased osteoclastogenesis, and IL-17A promotes OCL differentiation by inducing secretion of RANKL. Furthermore, it is known that RANKL is produced by various BM cells, including OBL, lymphocytes and adipocytes^26,36^. pOCL enter the BM from the circulation where they differentiate into multinucleated bone-resorbing OCL. Using flow cytometry, pOCL in BM were analyzed by their expression of RANK and macrophage colony stimulating factor 1 receptor (MCSF-R) as described before^37^. After OVX, the pOCL population, as well as OCL on the endosteal bone surface, increased significantly as expected in WT mice, and a tendency towards an increase was observed also in the KO mice. The same pattern was observed for RANKL expression on T cells and *Rankl* mRNA levels in cortical bone. This indicates that the KO mice, like the WT mice, have the potential to increase osteoclastogenesis after OVX, which could explain the loss of trabecular bone in KO mice. Thus, it is interesting to speculate whether also cortical bone mass would decrease in ovariectomized KO mice if longer time would pass between OVX and the termination of the experiment. Besides osteoclastogenesis, serum markers of bone turnover were analyzed. Changes in CTX-I and PINP reflect bone remodeling after OVX and both markers have previously been shown to increase up to two weeks after OVX^27^. Interestingly, and in contrast to WT mice, both CTX-I and PINP remained unchanged after OVX in KO mice, indicating decreased total bone turnover after OVX in mice lacking IL-17A. In addition, we did not observe any significant differences in the mineral apposition rate after OVX in either WT or KO mice, but both genotypes showed a tendency towards decreased bone formation at the diaphyseal endosteal bone surface.

Genetic depletion of IL-17A in mice has previously been shown to induce increased IL-17F secretion from splenocytes, however, whether and to what extent this compensatory mechanism affects bone homeostasis is unknown^38^. We used an *in vitro* approach to test the capacity of stimulated BM cells from both WT and KO mice to produce IL-17A and F. As expected, BM cells derived from KO mice did not secrete IL-17A, and IL-17F secretion from both WT and KO BM cells was very low even if a slight increase in IL-17F could be detected after stimulation with PMA and Ionomycin (Supplementary Fig. 8). In conclusion, while this culture system demonstrates the maximal capacity of BM cells to produce IL-17A and IL-17F after stimulation, it still remains elusive whether IL-17F can compensate for the loss of IL-17A in bone *in vivo*.

In contrast to the papers describing that loss of IL-17A signaling protects against bone loss after OVX^16,17,31^, Goswami *et al*. show that mice lacking the IL-17A receptor has decreased BMD after OVX compared to ovariectomized WT mice^20^. Additionally, they observe increased body weight of the IL-17RA KO mice, which further elevates after OVX. In accordance with the increased weight, the adipocyte marker leptin is elevated in the IL-17RA KO mice. They conclude that the bone destructive effect of depleted IL-17-signaling is due to upregulated central leptin expression, which is known to have anti-osteogenic effects^39,40^. Mechanistically, they hypothesize that the inhibitory effect of IL-17A on leptin levels helps to downregulate adipocyte differentiation in favor of OBL differentiation from MSC^41^. In line with results reported by Goswami *et al*., we observed that mice lacking IL-17A were not protected from OVX-induced trabecular bone loss; therefore, we investigated adipogenesis in our setting. Indeed, mice lacking IL-17A displayed increased adipogenesis as a response to estrogen depletion, depicted by increased percentage of body fat and leptin levels in serum. Similar to leptin, the adipokine adiponectin is also recognized for its anti-osteogenic role. It inhibits OBL proliferation and induces OBL apoptosis^42^. Interestingly, we found that *adiponectin* mRNA levels in BM of KO mice were elevated after OVX. BM adipocytes are known producers of RANKL and therefore also potential inducers of osteoclastogenesis^36^, and increased BM adipogenesis is associated with decreased trabecular BMD, a condition often seen in post-menopausal women^43–45^. In line with this, we observed an expansion of the adipocyte compartment in femur of ovariectomized KO mice possibly contributing to the differential effects of IL-17A on cortical and trabecular bone compartments.

Thus, our findings could explain the discrepancies seen between different models affecting IL-17 signaling in mice. Likely, short-term antibody treatments do not affect adipogenesis to the same extent as life-long deletion of the cytokine in transgenic mouse models. IL-17A is a known driver in many inflammatory diseases affecting bone, e.g. ankylosing spondylitis (AS), psoriatic arthritis (PsA) and RA. Interestingly, anti-IL-17A treatment with secukinumab has beneficial effects in patients with AS and PsA, but to lesser extent in patients with RA^46^. One possible explanation could be the different nature of the diseases; while RA is a bone destructive disease, AS and PsA are associated with both aberrant bone formation and bone resorption^47^. Based on our findings it is interesting to speculate how long-term treatment of patients with IL-17A/IL-17RA antibodies affects adipogenesis and bone homeostasis.

In summary, results from this study show that IL-17A is an important mediator of cortical but not trabecular bone loss after OVX. In addition, we show that IL-17A induces a two-sided effect on bone remodeling in the absence of estrogen; first by increasing osteoclastogenesis, and second by decreasing adipogenesis, which potentially leads to increased bone formation.

## Supplementary information

Supplementary Information.
