# Supplementary material for: Interleukin 17A: a Janus-faced regulator of osteoporosis
Source: Sci Rep. 2020 Mar 30;10:5692. doi: 10.1038/s41598-020-62562-2 (PMC7105470; doi:10.1038/s41598-020-62562-2)
Supplement: Supplementary file 1 — Supplementary Information. [file 41598_2020_62562_MOESM1_ESM.pdf]

Interleukin 17A: a Janus-faced regulator of osteoporosis

Scheffler JM[1,2], Grahnemo L[2], Engdahl C[2,3], Drevinge C[1,2], Gustafsson KL[2], Corciulo C[1,2], Lawenius L[2], Iwakura Y[5], Sjögren K[2], Lagerquist MK[2], Carlsten H[3], Ohlsson C[2,4], Islander U[1,2]

[1]Krefting Research Center, Department of Internal Medicine and Clinical Nutrition, [2]Centre for Bone and Arthritis Research, Department of Internal Medicine and Clinical Nutrition, [3]Centre for Bone and Arthritis Research, Department of Rheumatology and Inflammation Research, Institute of Medicine, Sahlgrenska Academy at University of Gothenburg, Gothenburg, Sweden; [4]Region Västra Götaland, Sahlgrenska University Hospital, Department of Drug Treatment, Gothenburg, Sweden; [5]Research Institute for Biomedical Sciences, Tokyo University of Science, Japan

*Corresponding author
Julia M Scheffler, PhD
Krefting Research Centre
Department of Internal Medicine and Clinical Nutrition
Sahlgrenska Academy
University of Gothenburg
Box 424
405 30 Gothenburg
Sweden

e-mail: julia.scheffler@gu.se
phone: +46-760-761908

Suppl. Fig. 1

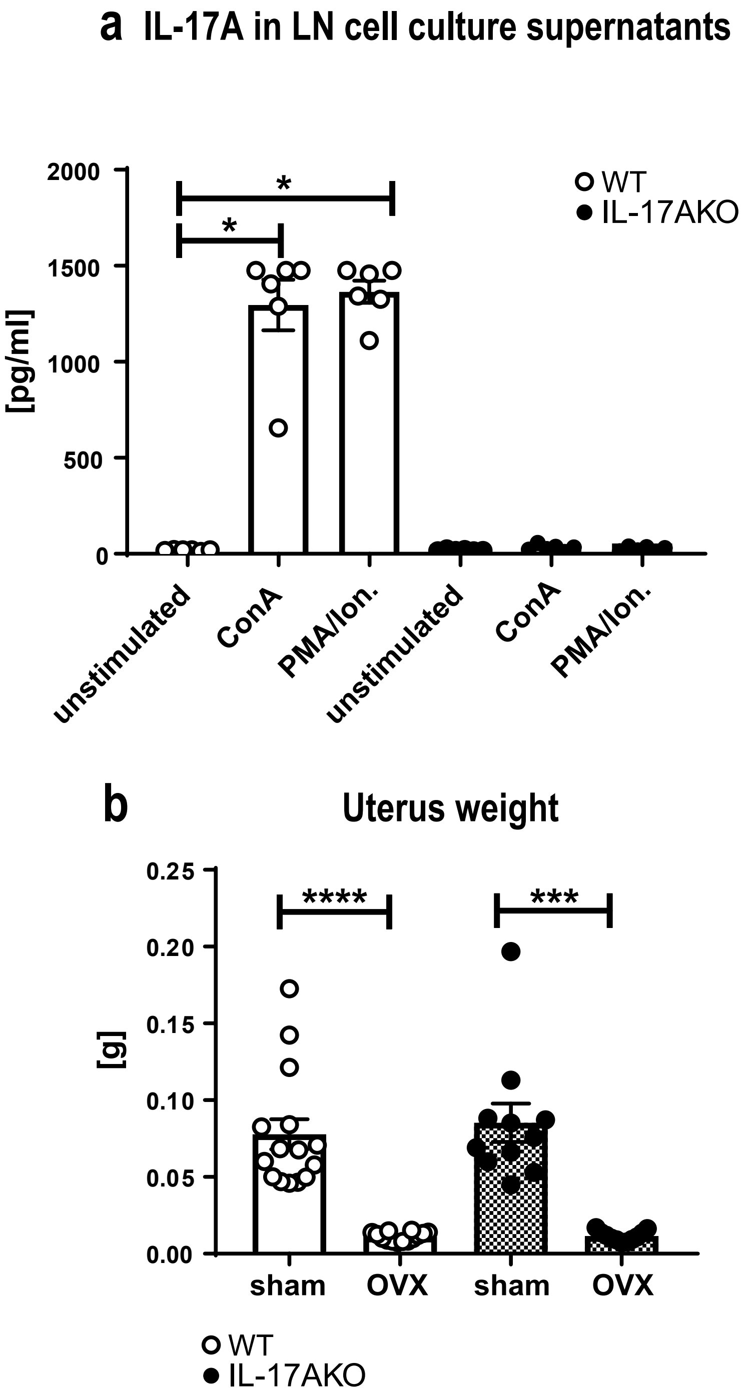

Suppl. Fig. 1: a) Interleukin (IL)-17A secretion from lymph node cell culture supernatants after stimulation with either Concanavalin A (ConA) or phorbol 12-myristate 13-acetate/ Ionomycin (PMA/Ion.) *in vitro*. n= 3 WT females and 2-3 WT males; 2-3 KO females and 3 KO males; *P < 0.05, Kruskal-Wallis test. **b)** Uterus weight of WT and KO mice 3 weeks after surgery. n= 15 WT sham females; 14 WT OVX females; 11 KO sham females; 11 KO OVX females. ***P < 0.001, ****P < 0.0001, Kruskal-Wallis test. Each dot represents one mouse.

Suppl. Fig. 2

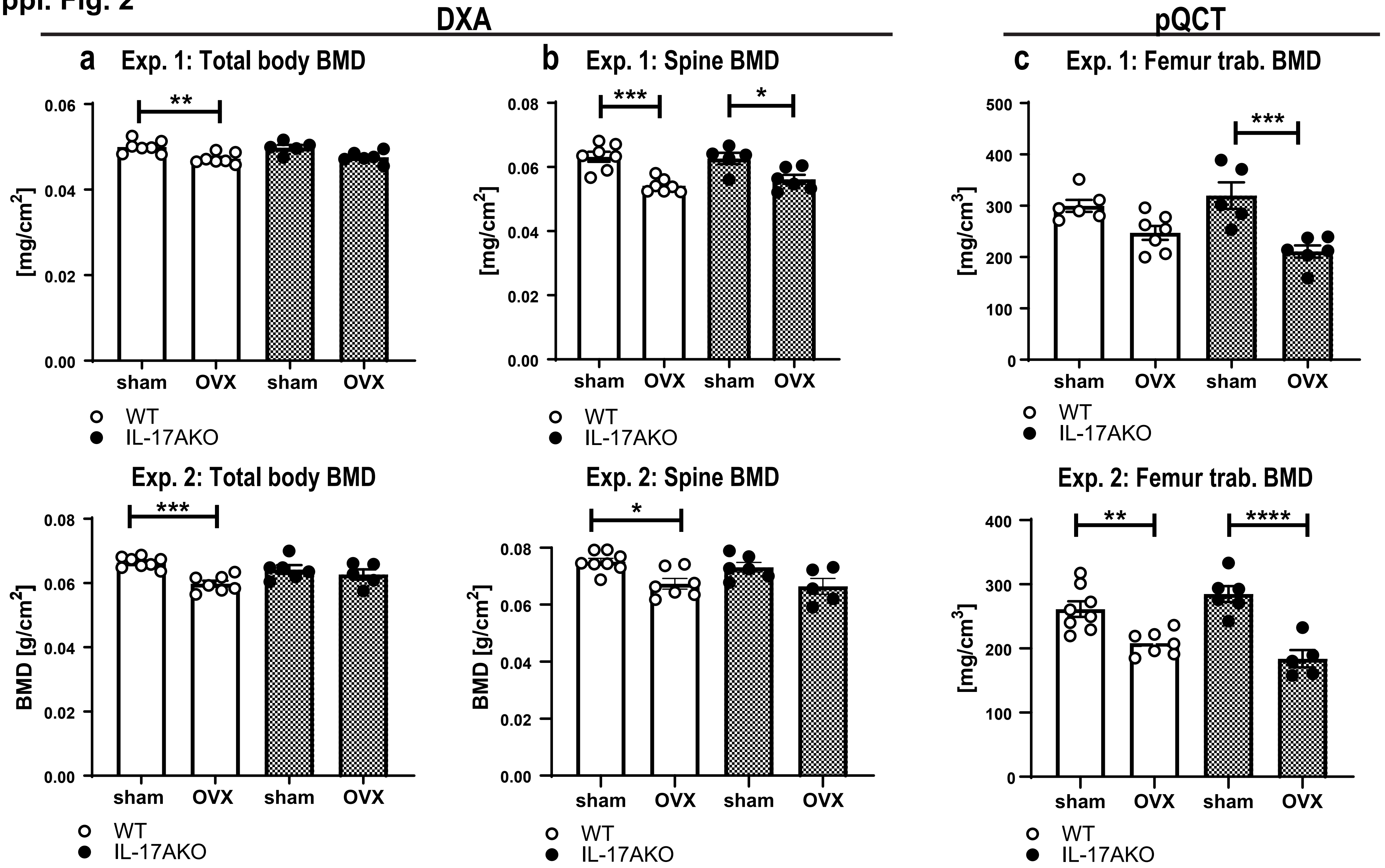

Suppl. Fig. 2: Results from two independent experiments for bone analyses: Dual-energy X-ray absorptiometry (DXA) for **a)** total body bone mineral density (BMD) and **b)** spine BMD, **c)** Peripheral Quantitative Computed Tomography (pQCT) for femur trabecular BMD. Each dot represents one mouse. n= 7-8 WT sham females; 7 WT OVX females; 5-6 KO sham females, 5-6 KO OVX females, *P < 0.05, **P < 0.01, ***P < 0.001, ****P < 0.0001, One-way ANOVA. Each dot represents one mouse

Suppl. Fig. 3

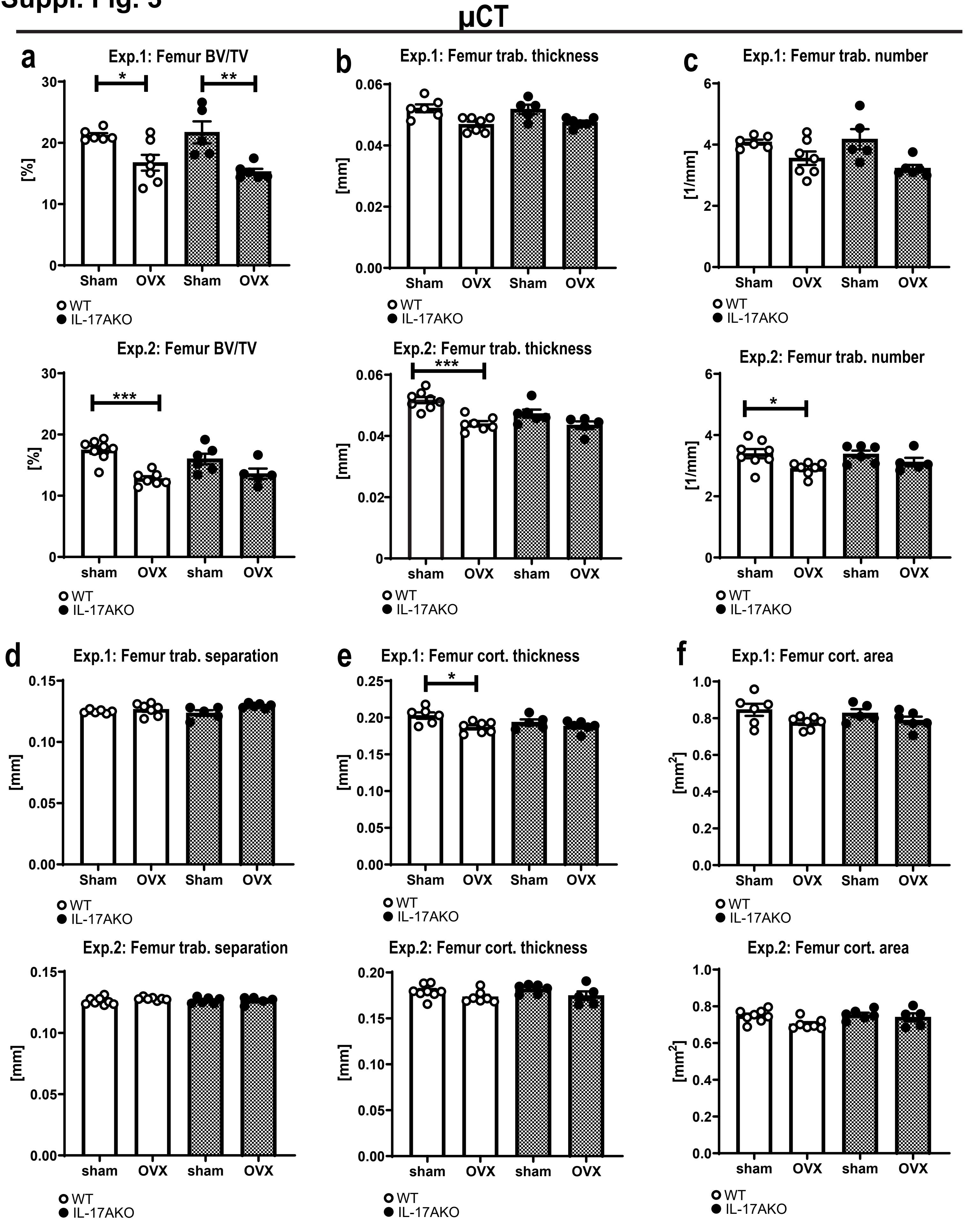

Suppl. Fig. 3: Results from two independent experiments using High-resolution microCT (µCT) for bone analyses are shown: **a)** femur BV/TV, **b)** femur trabecular thickness, **c)** femur trabecular number, **d)** femur trabecular separation, **e)** femur cortical thickness and **f)** femur cortical area. n= 7-8 WT sham females; 7 WT OVX females; 5-6 KO sham females, 5-6 KO OVX females, *P < 0.05, **P < 0.01, ***P < 0.001, One-way ANOVA. Each dot represents one mouse.

Suppl. Fig. 4

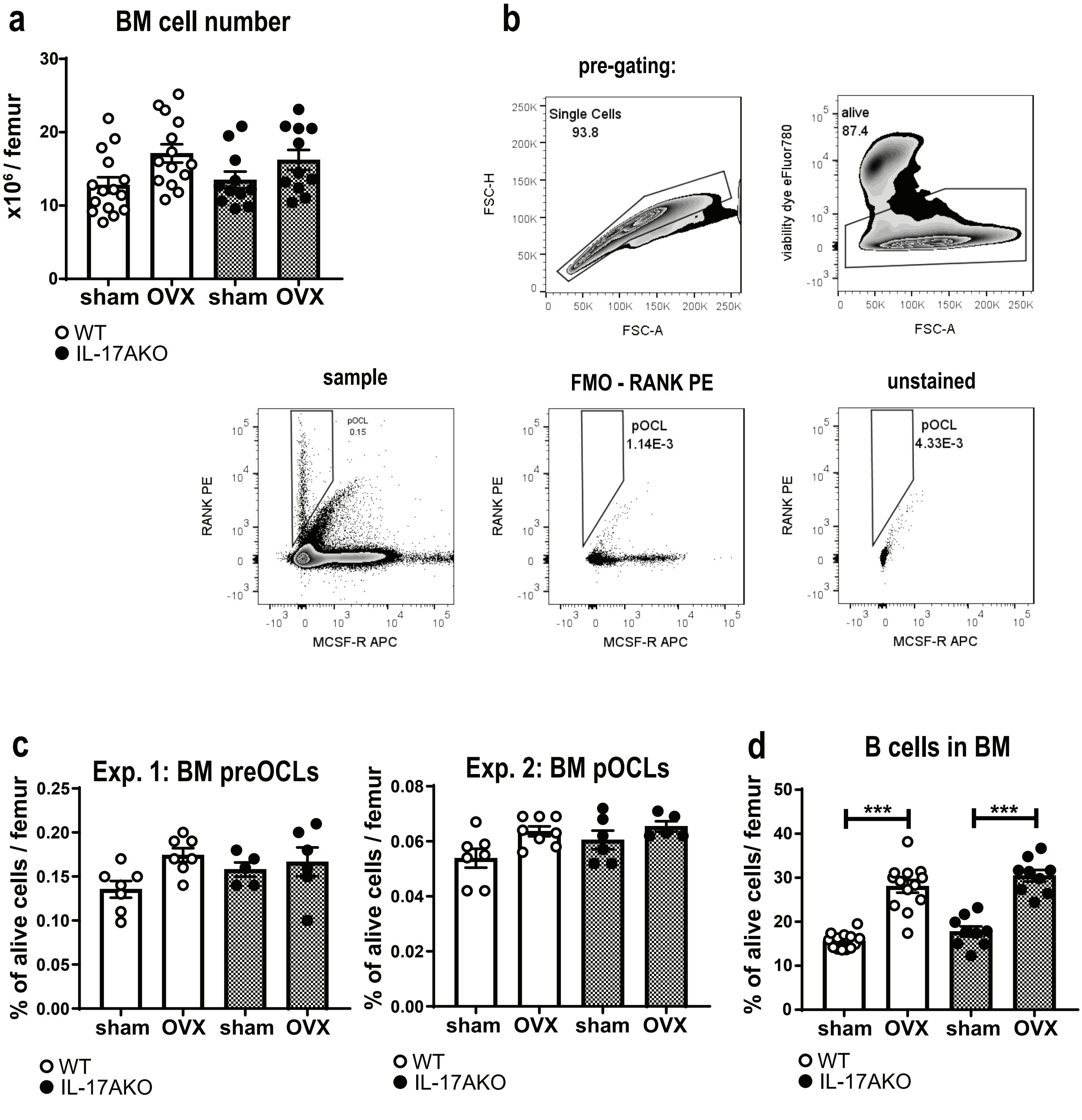

Suppl. Fig. 4: a) Femurs of female mice were dissected at termination (three weeks after surgery) and bone marrow (BM) cells were flushed out and counted. Numbers from one femur after lysis of erythrocytes are shown. n= 15 WT sham females; 14 WT OVX females; 11 KO sham females, 11 KO OVX females, One-way ANOVA. **b)** Gating strategy for pre-osteoclasts (pOCL). Cells were pre-gated for singlets and alive cells (negative for viability dye). $RANK^{pos}$ $MCSF\text{-}R^{neg}$ cells were gated as pOCLs according to FMO (Fluorescence minus one: RANK staining). Additionally, an unstained sample is shown. **c)** Results from two independent experiments for flow cytometry analysis of pOCLs in BM are shown. n= 7-8 WT sham females; 7 WT OVX females; 5-6 KO sham females, 5-6 KO OVX females, One-way ANOVA. **d)** B cells in BM were analyzed by flow cytometry and defined as $CD19^{+}$ cells. n= 15 WT sham females; 14 WT OVX females; 11 KO sham females, 11 KO OVX females, One-way ANOVA. Each dot represents one mouse.

Suppl. Fig. 5

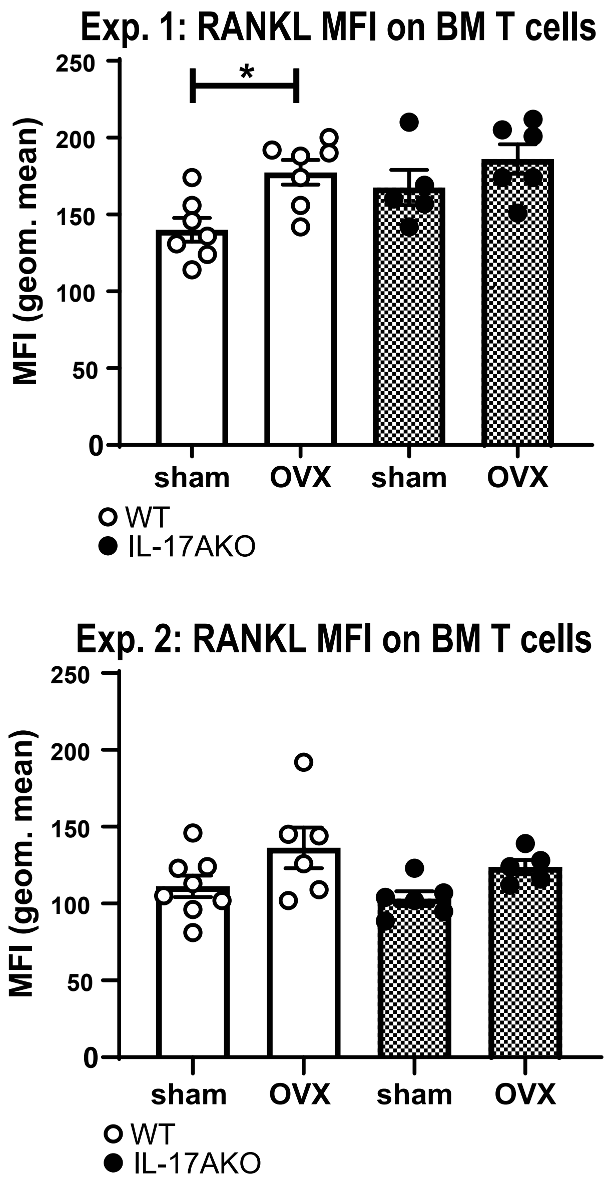

Suppl. Fig. 5: The geometric mean of RANKL fluorescence (mean fluorescent intensity (MFI)) on T cells. Each graph depicts an independent experiment. n= 7-8 WT sham females; 7 WT OVX females; 5-6 KO sham females, 5-6 KO OVX females, $^{*}P < 0.05$, One-way ANOVA. Each dot represents one mouse.

Suppl. Fig. 6

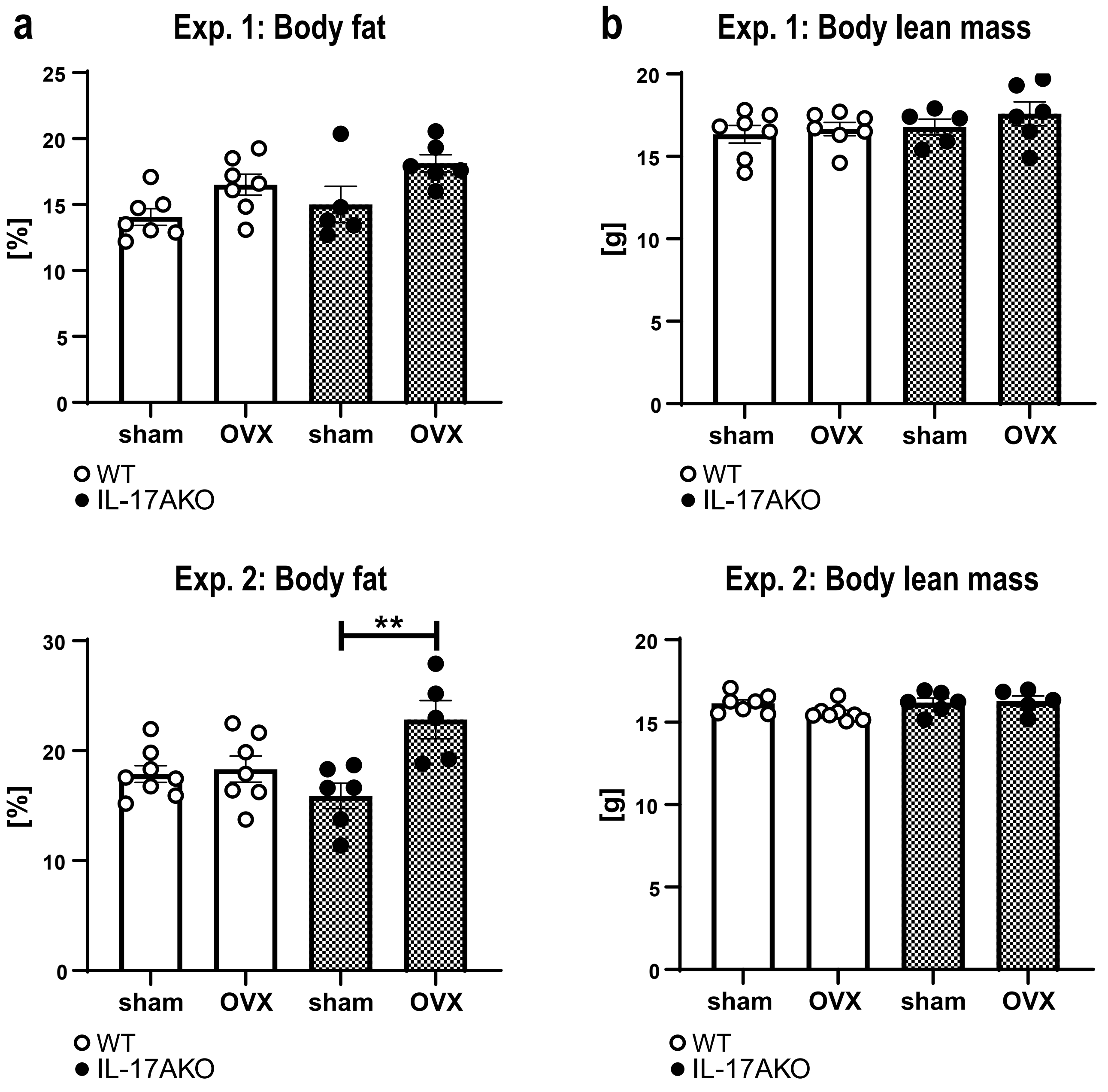

Suppl. Fig. 6: Results from two independent experiments using DXA are shown: **a)** percentage of body fat, and **b)** body lean mass. n= 7-8 WT sham females; 7 WT OVX females; 5-6 KO sham females, 5-6 KO OVX females, **P < 0.01, One-way ANOVA. Each dot represents one mouse.

Suppl. Fig. 7

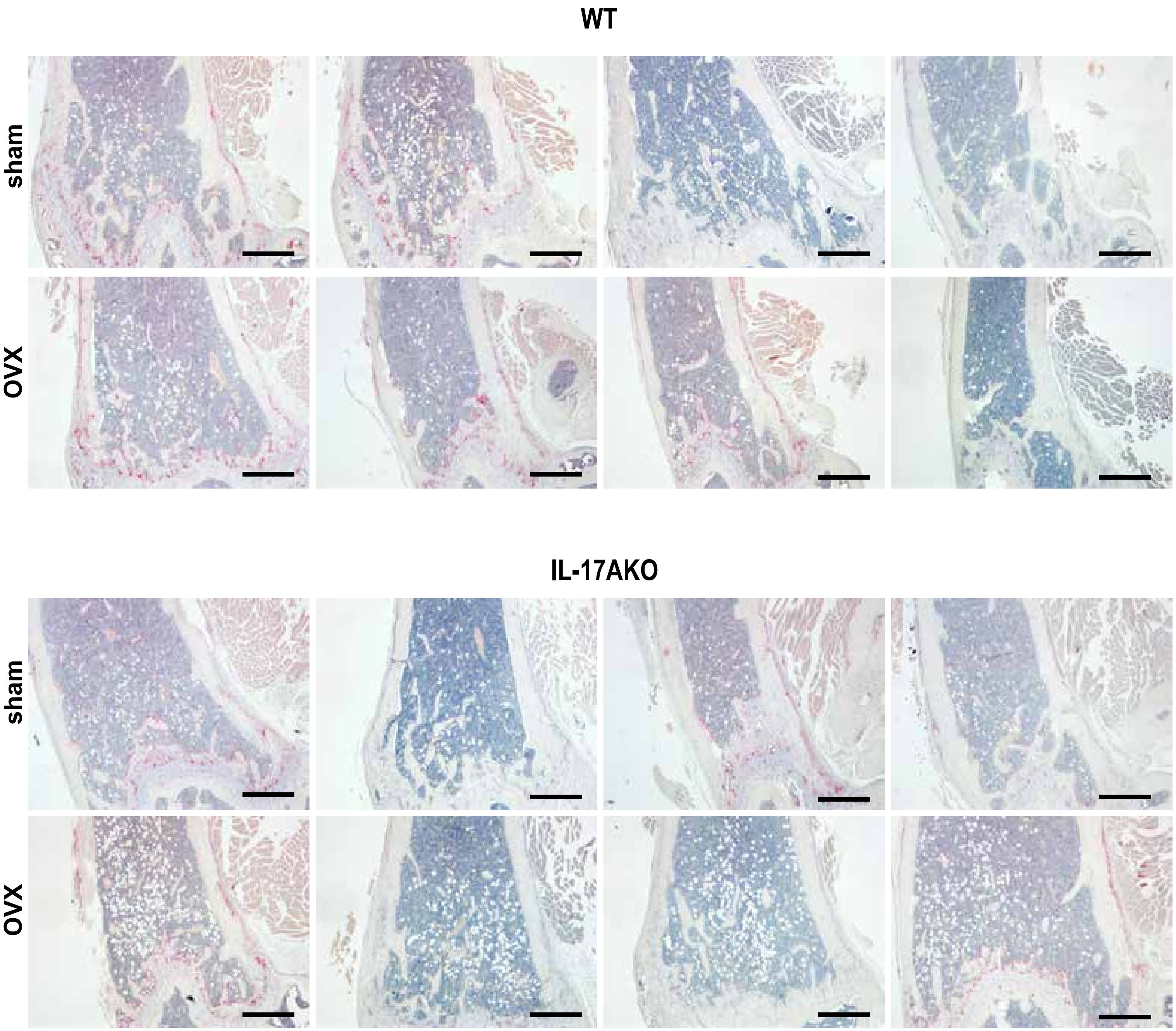

Suppl. Fig. 7: Paraffin sections of the epi- and metaphyseal regions of femur. Sections were stained with hematoxylin (blue) and TRAP (Tartrate-resistant acid phosphatase, red). Four representative images for each group are shown. Scale = 500µm.

Suppl. Fig 8

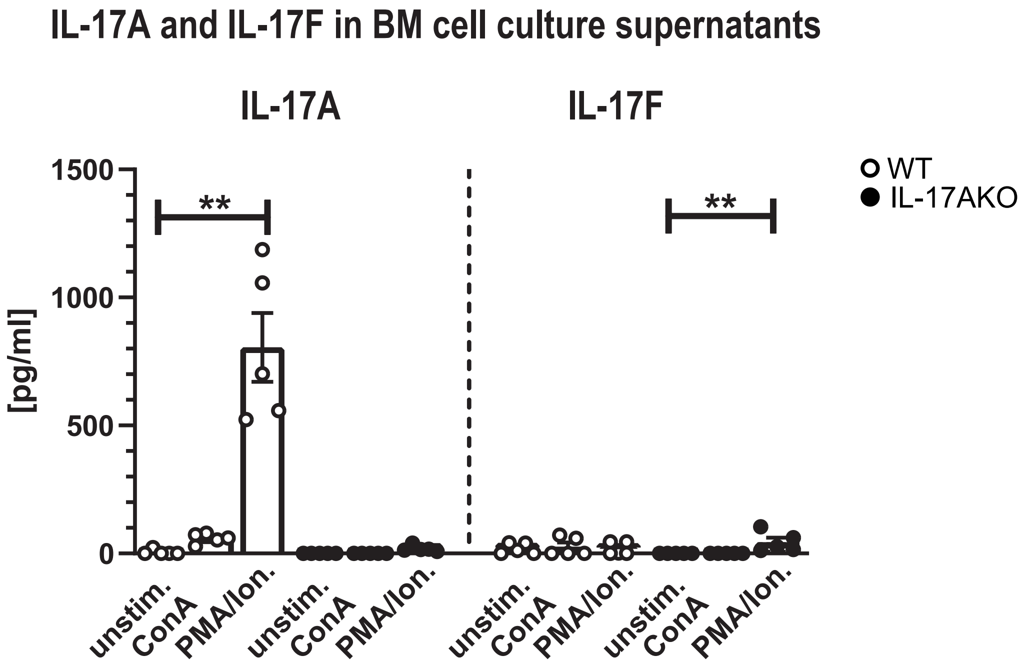

Suppl. Fig. 8: BM cells isolated from male and female WT and KO mice were stimulated with either Concanavalin A (ConA) or phorbol 12-myristate 13-acetate/ Ionomycin (PMA/Ion.) *in vitro*. IL-17A and IL-17F in the supernatant were measured after 48h with ELISA. n= 3 WT females and 2-3 WT males; 2-3 KO females and 3 KO males; $^{**}P < 0.01$, Kruskal-Wallis or One-way ANOVA test. Each dot represents one mouse.
